# The dual role of PGAM5 in inflammation

**DOI:** 10.1038/s12276-025-01391-7

**Published:** 2025-02-10

**Authors:** Yuxin Qi, Bhavana Rajbanshi, Ruihan Hao, Yifan Dang, Churong Xu, Wei Lu, Liming Dai, Bingjun Zhang, Xiaoling Zhang

**Affiliations:** 1 Department of Orthopedic Surgery, Xin Hua Hospital Affiliated to Shanghai Jiao Tong University School of Medicine, Shanghai, China; 2 Collaborative Innovation Centre of Regenerative Medicine and Medical BioResource Development and Application Co-constructed by the Province and Ministry, Guangxi Medical University, Nanning, China; 3 National Facility for Translational Medicine, Shanghai, China; 4 Department of Dermatology and Venereology, Tongji University School of Medicine, Shanghai, China; 5 School of Medicine, Shanghai University, Shanghai, China; 6 CAS Key Laboratory of Tissue Microenvironment and Tumor, Shanghai Institute of Nutrition and Health, University of Chinese Academy of Sciences, Chinese Academy of Sciences, Shanghai, China

**Keywords:** Apoptosis, Inflammatory diseases

## Abstract

In recent years, the focus on human inflammation in research has increased, with aging-related inflammation widely recognized as a defining characteristic of aging. Inflammation is strongly correlated with mitochondrial dysfunction. Phosphoglycerate mutase family member 5 (PGAM5) is a novel modulator of mitochondrial homeostasis in response to mechanical stimulation. Here we review the structure and sublocalization of PGAM5, introduce its importance in programmed cell death and summarize its crucial roles in the development and progression of inflammatory diseases such as pneumonia, hepatitis, neuroinflammation and aging. Notably, PGAM5 has dual effects on controlling inflammation: distinct PGAM5-mediated mitochondrial functions exhibit cellular heterogeneity, leading to its dual functions in inflammation control. We therefore highlight the double-edged sword nature of PGAM5 as a potential critical regulator and innovative therapeutic target in inflammation. Finally, the challenges and future directions of the use of PGAM5, which has dual properties, as a target molecule in the clinic are discussed. This review provides crucial insights to guide the development of intelligent therapeutic strategies targeting PGAM5-specific regulation to treat intractable inflammatory conditions, as well as the potential extension of its broader application to other diseases to achieve more precise and effective treatment outcomes.

## PGAM5 dual role in inflammation and mitochondrial health

This Review explores the role of the PGAM5 protein in inflammation and aging and investigates how PGAM5 affects mitochondrial function and inflammation. The authors review various experiments showing that PGAM5 can both promote and reduce inflammation by influencing processes such as mitochondrial fission and mitophagy, which are crucial for maintaining cell health. This research highlights that PGAM5 interacts with other proteins to regulate cell death and inflammation. The findings suggest that PGAM5 has a dual role: it can either worsen or alleviate inflammation depending on the context. This dual nature makes it a potential target for treating inflammatory diseases. Future research could focus on developing therapies that modulate PGAM5 activity to treat conditions such as arthritis or neurodegenerative diseases.

This summary was initially drafted using artificial intelligence, then revised and fact-checked by the author.

## Introduction

An excessive immunological response and proinflammatory factor overproduction may cause a pathologic response. Long-term proinflammatory factor activation can even accelerate aging; hence aging is also considered a consequence of inflammation^1^. Pattern recognition receptors are activated by damage-associated molecular patterns (DAMPs) released from damaged or dead cells, such as nucleic acids, adenosine triphosphate and mitochondrial contents, thereby activating cellular immune responses under physiological conditions^2^. Recent research has suggested that mitochondria are remnants of a proteobacterial lineage^3^ and that many of their components and metabolites (for example, mitochondrial (mt)DNA) serve as ligands for pattern recognition receptors and regulate cellular inflammation. Owing to the close link between inflammation and mitochondrial dysfunction, mitochondria play a crucial role in regulating the progression of inflammation.

Many mitochondrial proteins are involved in the inflammatory process. These proteins include (1) mitochondrial membrane channel proteins, which affect the permeability of the mitochondrial membrane. For example, the apoptotic pores formed by BCL-2-associated X protein (BAX), B cell lymphoma-extra large (Bcl-xL) and BCL-2 antagonist/killer (BAK) release mtDNA into the cytoplasm, which activates cGAS–STING signaling, induces the sustained release of inflammatory cytokines and drives the inflammatory response. (2) Mitochondrial quality control-related proteins are also involved. For example, PINK1-mediated mitophagy removes damaged mitochondria and inhibits inflammation^4^. In addition, the excessive fission induced by Drp1 may damage mitochondria and increase reactive oxygen species (ROS) production, promoting inflammatory responses^5^. These mitochondrial proteins have frequently been explored in studies to elucidate the specific mitochondrial functions involved in inflammation, such as PINK1-regulated mitophagy and Drp1-regulated mitochondrial fission. Given the diversity of mitochondrial functions, identifying the proteins that facilitate the integration of these diverse processes is crucial. These proteins assist in processes such as mitochondrial quality control, programmed cell death and mitochondria-mediated signaling, which regulate the inflammatory response. At this stage, phosphoglycerate mutase family member 5 (PGAM5) has emerged as a critical mediator of multiple mitochondrial biological functions and is pivotal for the development of mitochondria and inflammation.

PGAM5, a crucial mitochondrial protein, possesses a GTPase activator and serine/threonine protein phosphatase activities^6^. Under normal physiological conditions, PGAM5 interacts with several mitochondrial, anti-apoptotic, or cytoplasmic proteins and regulates mitochondrial morphology and function through its phosphatase activity^7,8^. However, under pathological conditions, PGAM5 interacts with the intracellular proteins Bcl-xL, NDPK-B and Drp1. It leads to mitochondrial dysfunction, which manifests as excessive mitochondrial fission and accelerated mitophagy, and it activates biological processes such as cell death and stress^6^. An increased understanding of the role of PGAM5 has led to the hypothesis that it is strongly associated with inflammation. Recent research has shown that PGAM5 is involved in multiple forms of inflammation. Li et al. reported that PGAM5 induces mtDNA release and activates the cGAS‒STING pathway by dephosphorylating the apoptotic protein BAX, thereby accelerating acute kidney injury (AKI)-induced inflammation^9^. In contrast, in brain tissue, decreased PGAM5 expression does not reduce inflammation, but rather exacerbates the inflammatory response^10^. For example, in a rat model of permanent focal cerebral ischemia, downregulated PGAM5 inhibits Drp1-mediated mitophagy and then exacerbates mitochondria-dependent injury^11^. Similarly, in carbonyl cyanomethyl chlorophenylhydrazone (CCCP)-treated cells, PGAM5 exerts a neuroprotective effect by regulating mitophagy^12^. Furthermore, specific levels of PGAM5 expression can promote organismal health and potentially impede the onset of premature aging^13^. Conversely, inadequate PGAM5 expression results in mitochondrial dysregulation, exacerbating the inflammatory response and accelerating the aging process^14,15^. Therefore, PGAM5 has two distinct functions: one that negatively impacts diseases such as inflammation and other anti-inflammatory and protective roles. We hypothesized that the function of PGAM5 differs during different inflammatory responses, which results in PGAM5 displaying both proinflammatory and anti-inflammatory functions (Table 1).

**Table 1:** Dual roles of PGAM5 in inflammation.

Effects	Biological processes regulated by PGAM5	Related inflammatory diseases	Type of cell line	References
Proinflammatory	Mitochondrial fission, necroptosis	Acute liver injury	Hepatocytes	^15^
	Apoptosis, mitophagy	Hepatic ischemia–reperfusion injury	Hepatocytes	^31,100^
	Inflammasome activation	TBI	Microglia	^66^
	PGAM5–NRF2 axis	Ischemic stroke	Rat brain microvascular endothelial cells (rBMECs)	^95^
	Apoptosis	AKI	Renal tubular epithelial cells	^9^
	Necroptosis	Myocardial ischemia–reperfusion injury	Mouse embryonic fibroblasts (MEFs), cardiomyocytes	^101^
	Mitochondrial fission	Cell senescence	Human retinal epithelial cells	^13^
Anti-inflammatory	Mitophagy, mitochondrial biogenesis, pyroptosis	TBI	Neurons	^97^
	Mitochondrial fission	Cellular senescence	Oocytes	^14^
	TBK1/IRF3-dependent antiviral responses	Vesicular stomatitis virus infection	MEFs	^102^
	Apoptosis	Myocardial ischemia–reperfusion injury	Cardiomyocytes	^84^
	Oxeiptosis	Respiratory inflammation caused by viruses	MEFs	^67^
	Mitophagy	Osteoarthritis	Chondrocytes	^103^

**Fig. 1:**
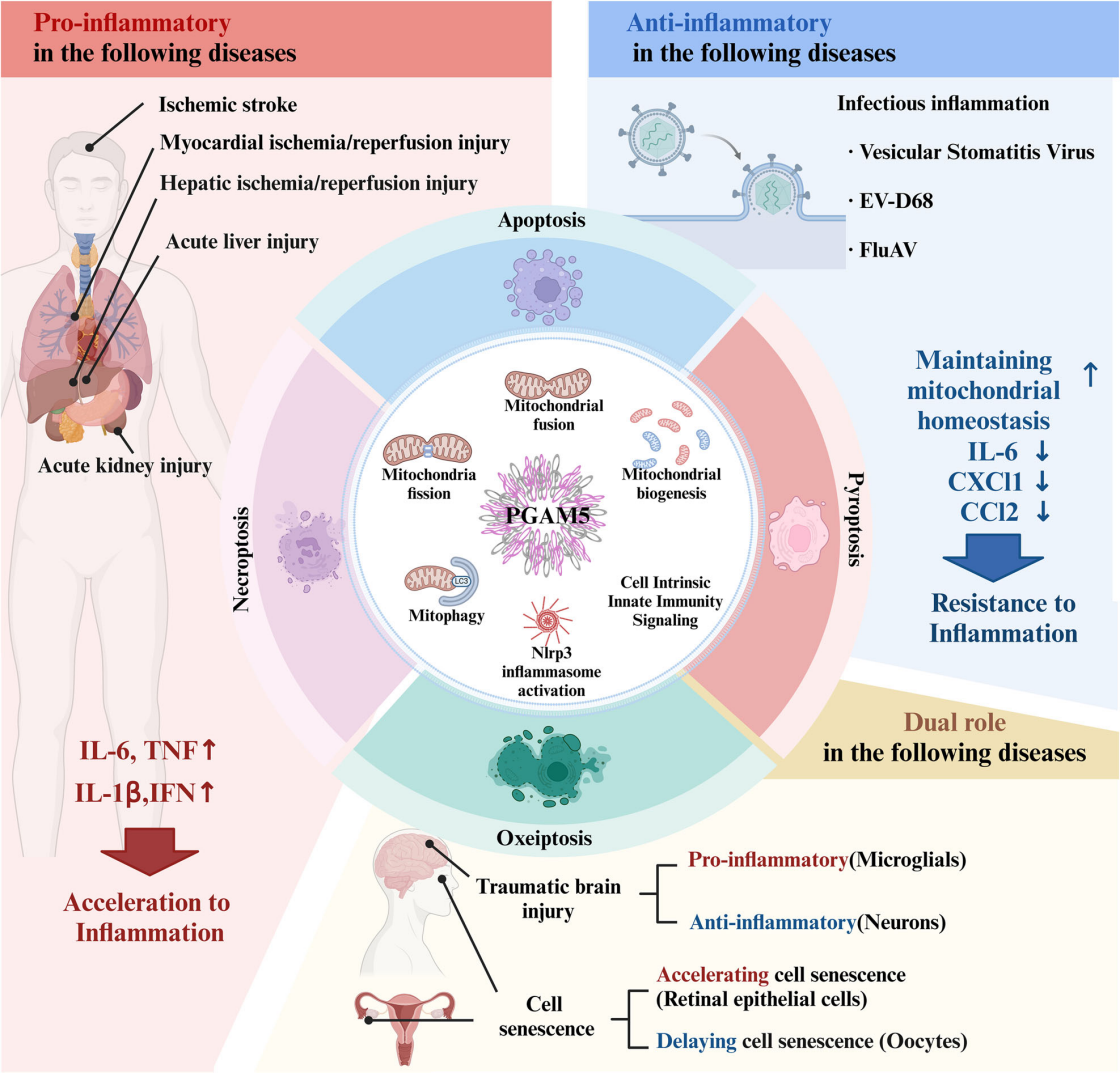
The dual roles of PGAM5 in the development of inflammation. PGAM5 predominantly regulates various mitochondrial functions and Nlrp3 inflammasome activation in cells, as well as the activation of innate immune pathways. In addition, PGAM5 is involved in various types of programmed cell death, including apoptosis, necroptosis, pyroptosis and oxeiptosis. In diverse types of inflammation, PGAM5 promotes the secretion of various proinflammatory cytokines to accelerate inflammation. However, it is also an anti-inflammatory agent that can attenuate inflammation and contribute to the maintenance of mitochondrial quantity and quality, thereby maintaining cellular homeostasis in infectious diseases. Even during traumatic brain injury and cellular senescence, PGAM5 has both anti-inflammatory and proinflammatory properties.

In the past decade, many reviews have focused on the biological activities of PGAM5 and the roles of PGAM5 in neurological disorders. However, the physiological and pathological roles of PGAM5 have been updated with recent research progress, and a new function of PGAM5 in oxeiptosis has been identified and validated. Therefore, a new and comprehensive review of the heterogeneous cell functions and disease specificity of PGAM5 is needed to systematically summarize the biological properties of PGAM5 and detail its physiological role in inflammation. This review summarizes the functions of PGAM5 in inflammation (Fig. 1) and provides novel insights and strategies for future research on PGAM5 and innovative approaches to drug development for relevant diseases.

## Subcellular localization and regulation of PGAM5 expression

### Subcellular localization of PGAM5

PGAM5 is widely distributed in various tissues, including the heart, brain and kidney^16,17^. Elucidating the mechanism of action of PGAM5 requires its subcellular localization to be discerned. PGAM5 dephosphorylates cytoplasmic proteins such as ASK1 (ref. ^18^), Drp1^19,20^, NFAT^21^ and FUNDC1 (ref. ^22^), indicating its localization in the cytoplasmic lysosome. PGAM5 is generally known to be located on the inner mitochondrial membrane (IMM) based on its cofractionation with markers of the IMM in isolated mitochondria^6,16,23^. Considering the low energy barrier for protein translocation between the mitochondria-associated endoplasmic reticulum (ER) membrane and mitochondria, PGAM5 conceivably shuttles between these organelles, akin to other proteins such as FKBP38 and BCL-2, during mitophagy^24^. This hypothesis warrants further investigation in future studies.

In addition, a form of PGAM5 cleaved by PARL was detected in the cytosol. PGAM5 is anchored to the IMM via its transmembrane domain, and part of this domain interacts with PARL during mitochondrial dysfunction, leading to PGAM5 cleavage^16^. This process occurs when the mitochondrial membrane potential (Δ*Ψ*_m_) is lost, after which the cleaved PGAM5 can be released from the mitochondria through outer mitochondrial membrane (OMM) rupture mediated by proteasomes^25^. Furthermore, the function and localization of PGAM5 are regulated by syntaxin 17 (Stx17), which is critical for sustaining normal mitochondrial function and engaging in mitochondrial clearance^23^.

Cleaved PGAM5 is distinctly distributed within the cell and is present both in the cytoplasm and nucleus^12,26^. During CCCP-induced mitophagy, a portion of cleaved PGAM5 relocates to the nucleus. In this context, PGAM5 modulates the phosphorylation status of the nuclear proteins SRm160 and SR. Given the pivotal roles of SRm160 and SR in mRNA metabolism^26^, PGAM5 conceivably orchestrates cellular responses to mitochondrial stress, which could affect both post-transcriptional and pretranslational processes.

### Regulation of PGAM5 expression

PGAM5 is divided into three main domains: the N-terminal mitochondrial targeting sequence, the C-terminal PGAM phosphatase structural domain and various functional motifs in the middle. The N-terminal region contains the conserved WDXNWD motif, which is the basis for the formation of large multimers and enhances enzyme stability. In addition, the N-terminal mitochondrial targeting sequence is a transmembrane helix that can be targeted to the mitochondrial membrane^17^. Amino acids 24–25 are the cleavage site for PARL, and after PARL action, part of the PGAM5 fragment is released into the cytoplasm^16^. PGAM5 shares a conserved PGM catalytic center at its C-terminus to perform its phosphatase function, and the highly plastic catalytic center interacts with phosphate molecules to form three structural modes: (1) a phosphate-free form (apo), (2) a phosphate-bound ‘on’ state (on) and (3) a phosphate-bound ‘off’ state (off)^27,28^. The α4–β5 loop, β6 strand and C-terminal tail from one molecule are packed against the corresponding region in the other protomer, which participate in intermolecular interactions to form a dimer that lacks the C-terminal tail (residues 29–278 and 81–278) and is predominantly present as monomeric forms, suggesting that the C-terminal tail plays an important role in stabilizing this assembly^27^. However, the PGAM5 dimer is hypoactive. The formation of a multimer structure with a WDPNWD motif is critical to stimulate the catalytic domain and achieve maximal phosphatase activity^27^. The structure of PGAM5 is complex and not yet fully understood, which is the basis for its functional diversity. Through different upstream and downstream pathways, it regulates mitochondrial morphology and function.

The upstream regulators of PGAM5 are classified into gene-level and protein-level regulators. Gene-level regulation involves the regulation of PGAM5 mRNA or protein expression by modulating transcription and translation. Protein-level regulation involves regulating PGAM5 function through epigenetic modifications, protein cleavage and localization and recruitment (Table 2).

**Table 2:** Regulation of PGAM5 expression.

	Gene	Function	Cell type	References
**Gene level**	Sp1	Binds to the PGAM5 promoter and promotes PGAM5 transcription	HK2 cells	^29^
	Stat5	Binds to the PGAM5 promoter and promotes PGAM5 transcription	N2A neurons; HEK293 cells	^39^
	miR-21-5p	Specifically binds to the 3′ untranslated region of PGAM5-WT and negatively regulates PGAM5 expression to inhibit mitophagy	AEC-II cells	^85^
	miR-330	By inhibiting PGAM5, miR-330 can downregulate mitophagy by inhibiting the PGAM5-induced dephosphorylation of Drp1	Hepatocytes; primary neurons	^11,98^
**Protein level**	SIRT2	Deacetylated PGAM5	Hepatocellular carcinoma	^30^
	MARCH2	Interacts directly with PGAM5 and promotes the ubiquitination and degradation of PGAM5 by attaching K48-linked polyUb to the PGAM5 K88 and K141 sites	Cardiomyocytes	^32^
	XIAP	An E3 ubiquitin ligase that inhibits PGAM5L-mediated cell death by attaching K48-linked polyUb to PGAM5	HEK293 cells	^33^
	RNF5	Degradation of PGAM5 mediated by K48-linked ubiquitination	Hepatocytes	^31^
	PARL	Cleavage of PGAM5 results in the release of the C-terminal portion of PGAM5 from the mitochondrial membrane	Cortical neurons	^16,34^
	OMA1	The enzyme binds to SLP2 and cleaves PGAM5 in depolarized mitochondria	MEFs	^35^
	Stx17	CHD is responsible for binding to the transmembrane structural domain of PGAM5 and regulates the localization of the mitochondrial phosphatase PGAM5 to promote dephosphorylation of Drp1 and mitochondrial fission	293T cells HeLa cells	^23^
	KEAP1	KEAP1 interacts with full-length PGAM5 and controls its degradation in the cytoplasm to inhibit mitophagy	PC6 cells; cortical neurons; HEK293 cells HeLa cells	^104^
	TIPE3	TIPE3 recruits PGAM5 to promote BAX and Drp1 accumulation at the mitochondrial cristae, which mediates mitochondrial outer membrane permeabilization, cristae remodeling, mitochondrial fragmentation and apoptosis	Head and neck squamous cell carcinomas (HNSCC) cells	^36^
	Bcl-xL	Under hypoxic conditions, Bcl-xL interacts with and inhibits PGAM5 to prevent dephosphorylation of the FUNDC1 Ser13 site, which activates hypoxia-induced mitophagy	HeLa cells	^105^

At the gene level, miR-330 strongly and complementarily pairs with the 3′ untranslated region of the PGAM5 mRNA and exhibits a high degree of interspecies conservation^22,23^. This process resulted in miR-330 downregulating PGAM5 expression in primary neurons and hindering PGAM5-mediated mitophagy, thereby exacerbating the development of cerebral ischemia^23^. Similarly, in hepatic ischemia–reperfusion injury, the downregulation of miR-330 fails to reduce PGAM5 levels, leading to increased mitophagy^22^. A recent study revealed an interaction between Sp1 and the PGAM5 promoter via chromatin immunoprecipitation analysis, suggesting that Sp1 initiates the expression of PGAM5 and activates the mitochondrial division process^29^.

At the protein level, SIRT2 activates malic enzyme 1 (ME1) activity by deacetylating PGAM5, thereby promoting lipid synthesis and hepatocellular carcinoma (HCC) cell proliferation^30^. RNF5 degrades PGAM5 through K48-linked ubiquitination, inhibiting the activation of apoptosis-regulated kinase 1 (ASK1) and its downstream c-Jun N-terminal kinase (JNK)/p38 (ref. ^31^). This process ultimately inhibits the inflammatory response and apoptosis in hepatic ischemia‒reperfusion injury. Similarly, MARCH2 directly interacts with PGAM5 to promote its ubiquitination and degradation by attaching K48-linked polyUb to the K88 and K141 sites of PGAM5 (ref. ^32^). This process reduces the coaggregation of PGAM5 and MAVS, inhibiting the activation of Nlrp3 inflammatory vesicles and cardiomyocyte pyroptosis^32^. Current studies of the regulation of the PGAM5 protein focus on its acetylation and ubiquitination, such as by SIRT2 (ref. ^29^), XIAP^33^, RNF5 (ref. ^31^) and MARCH2 (ref. ^32^), and its cleavage, localization and recruitment involving PARL^16,34^, OMA1 (ref. ^35^), Stx17 (ref. ^23^) and TIPE3 (ref. ^36^). PGAM5 competes with PINK1 for PARL-mediated cleavage, resulting in the cleavage of the C-terminus of PGAM5 by PARL^16,34^. This cleavage maintains PINK1 stability and regulates mitophagy. Furthermore, the NT region of TIPE3 binds to PGAM5, which in turn promotes the accumulation of BAX and Drp1 at mitochondrial cristae^36^. This process mediates mitochondrial outer membrane permeabilization, cristae remodeling, mitochondrial fragmentation and apoptosis.

## Biological functions of PGAM5

### PGAM5 regulates mitochondrial quality control

#### Regulation of mitochondrial dynamics by PGAM5

**Fig. 2:**
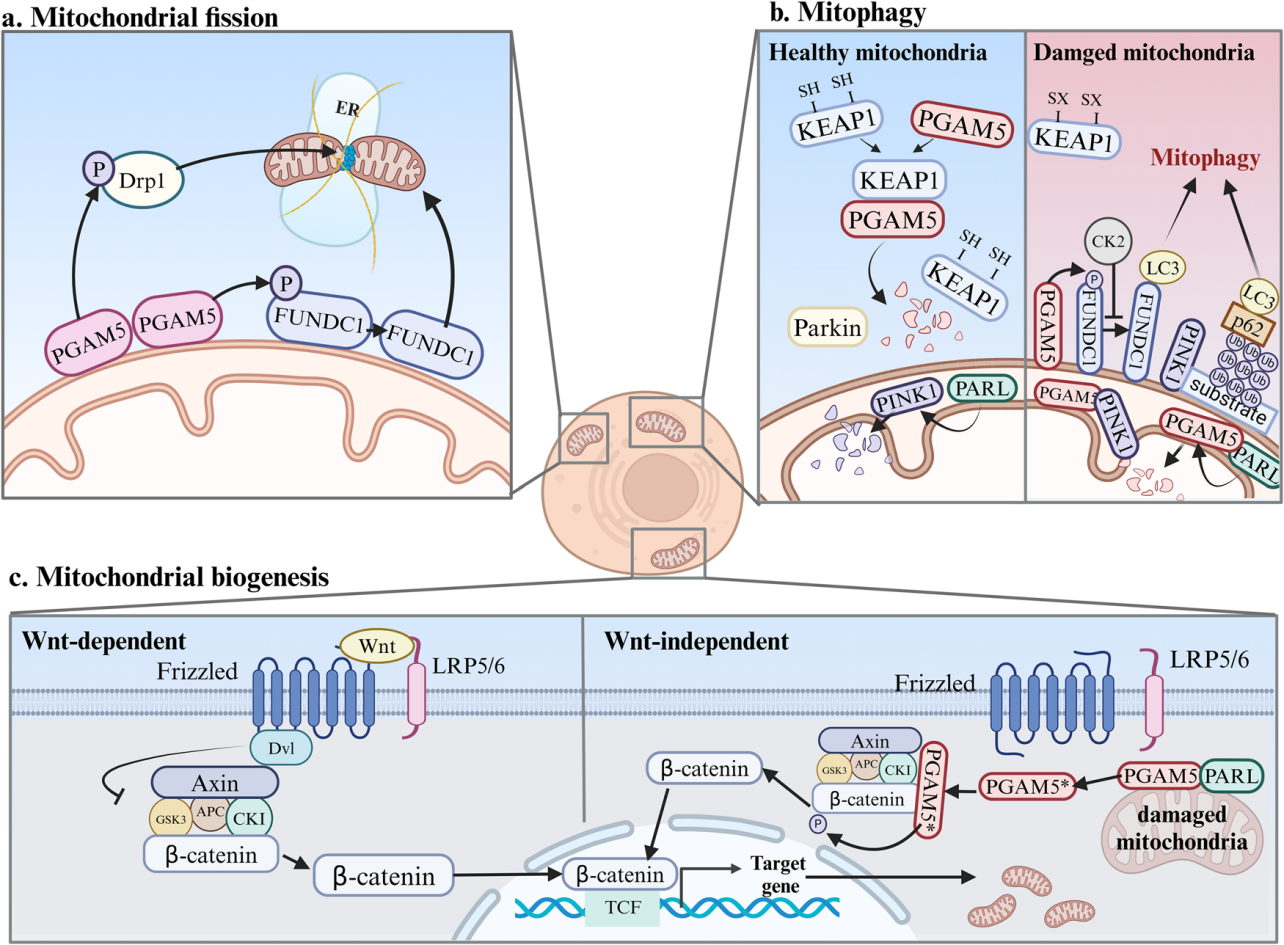
The regulation of mitochondrial quality control by PGAM5. **a** PGAM5 dephosphorylates Drp1 and facilitates its recruitment to mitochondria, thereby promoting mitochondrial fission and eliminating damaged mitochondrial segments via interactions with the ER and F-actin. **b** Left: in healthy mitochondria, PINK1 is translocated to the mitochondrial inner membrane and degraded by PARL, whereas PGAM5 is degraded by unoxidized KEAP1 before its translocation to the mitochondria. Right: in damaged mitochondria, PGAM5 replaces PINK1 as the substrate for PARL cleavage, resulting in the accumulation of PINK1 on the mitochondrial outer membrane. PINK1 recruits Parkin to ubiquitinate Parkin substrates, triggering mitophagy through the formation of autophagosomes and subsequent engulfment of mitochondria. In addition, PGAM5 can activate receptor-mediated mitophagy, such as FUNDC1-mediated mitophagy. **c** Damage to mitochondria causes PARL to cleave PGAM5, discharging it into the cytoplasm. The cleaved form of PGAM5 dephosphorylates β-catenin and induces its translocation to the nucleus, where it regulates the transcription of mitochondrial biogenesis-related target genes. Increased levels of the cleaved form of PGAM5 promote mitochondrial biogenesis. Notably, this mechanism does not involve the Wnt/β-catenin signaling pathway (left). Created with BioRender.com.

Cells must constantly adjust the balance between mitochondrial fission and fusion to respond to environmental fluctuations, ensuring the maintenance of mitochondrial morphology and function throughout biological processes, which is known as mitochondrial dynamics. Mitochondrial fission is indispensable for the induction of Drp1 (ref. ^37^). Several studies have shown that PGAM5 activates Drp1 by dephosphorylating Ser637 and phosphorylating Ser616. Activated Drp1 is then recruited and translocated to the mitochondrial membrane^15,38^. Consequently, increased oligomerization of Drp1 induces signaling at ER–mitochondria contact sites, resulting in the polymerization of F-actin (muscle actin fibers). This process promotes mitochondrial fission by causing the ER to engulf damaged mitochondria^39^. The function and localization of PGAM5 are regulated by the mitochondrial-associated membrane protein Stx17 (ref. ^23^). In healthy cells, the C-terminal hydrophobic domain (CHD) of Stx17 binds to the transmembrane structural domain of PGAM5, regulating the localization of the mitochondrial protein phosphatase PGAM5 to promote Drp1 dephosphorylation and mitochondrial fission. In response to PINK1/Parkin-mediated mitophagy, Stx17 releases PGAM5, promoting the dephosphorylation of FUNDC1 in response to mitochondrial autophagic stress^23^. In addition to its direct effect on Drp1, PGAM5 dephosphorylates FUNDC1, which induces the mitochondrial localization of Drp1 and mitochondrial fission^22^. These studies indicate that Drp1, a key factor in mitochondrial fission, regulates this process (Fig. 2a). These findings also reveal that PGAM5 is necessary for mitochondrial fission. However, excessive mitochondrial fission results in mitochondrial miniaturization^40^, which can cause adverse effects such as a important reduction in the mtDNA copy number, mitochondrial dysfunction and the generation of harmful ROS via the electron transport chain. In patients with Huntington’s disease, the mutant Huntington’s disease protein (mHtt) can bind to Drp1 and increase its activity, leading to excessive mitochondrial fragmentation and distribution irregularities, which in turn cause defects in the mitochondrial axonal transporter that ultimately lead to synaptic degeneration^41^.

Mitochondrial fusion enables the formation of novel mitochondria, and this process exchanges mitochondrial contents such as mtDNA from senescent or damaged mitochondria^42^. This exchange helps preserve mtDNA function and the integrity and copy number of mtDNA, and protects the cell from senescence^43^. Mitofusin 1/2 (Mfn1/2) and OPA1 play vital roles in regulating mitochondrial fusion and Mfn2 dephosphorylation^44^. Using *Drosophila* genetic models, researchers have shown that the Mfn2 counterparts Marf and dPGAM5 are components of the same biological pathway^45^. Moreover, PGAM5 was observed to bind to and dephosphorylate Mfn2 through co-immunoprecipitation experiments and stimulated mitochondrial network formation^45^. These findings indicate that PGAM5 acts as a dephosphatase for Mfn2, a mitochondrial fusion regulator, and contributes notably to the process of mitochondrial fusion.

#### Mitochondrial biogenesis is regulated by PGAM5

Mitochondrial biogenesis is crucial for preserving the quantity and quality of mitochondria, as well as for shifting irreversible cellular damage toward homeostatic recovery^46^. The response of cells to Δ*Ψ*_m_ loss triggered by CCCP treatment modulates the Wnt/β-catenin signaling pathway and promotes mitochondrial biogenesis^47^. Thus, in addition to its roles in growth, development and cell differentiation, the Wnt/β-catenin pathway has been reported to activate mitochondrial biogenesis. PINK1-mediated mitophagy leads to PGAM5 translocation from the mitochondria to the cytoplasm under pathological conditions. Axin, a core component of the β-catenin destruction complex, binds to β-catenin and PGAM5 via a separate structural domain, allowing PGAM5 to approach and dephosphorylate β-catenin, driving Wnt signaling and compensatory mitochondrial biogenesis^47,48^. The PGAM5–β-catenin axis represents a novel cell-intrinsic method of activating the Wnt/β-catenin pathway that acts independently of extrinsic Wnt ligands and represents a way to replenish the mitochondrial pool by activating Wnt/β-catenin signaling (Fig. 2c).

In addition, PGAM5-mediated regulation of mitochondrial biogenesis is associated with upregulated expression of PGC-1α, NRF1 and TFAM in a hypoxia/reoxygenation model^49^. The transcriptional coactivator PGC-1 α is a crucial regulator of mammalian mitochondrial biogenesis^50^, and its activation of the PGC-1 α-1/2–TFAM pathway was observed in a study of songorin therapy for heart failure. The interaction between PGC-1α and NRF2 was enhanced by the pharmacological intervention in lipopolysaccharide (LPS)-induced mice, resulting in synergistic activation of the NRF2/ARE and NRF1 pathways^17^. NRF1 activates TFAM, which participates directly in mtDNA replication to precisely control the mitochondrial mass and abundance^17^. PGAM5 is cleaved during mitophagy in human induced pluripotent stem cell-derived endothelial progenitor cells, which activates the transcription of PGC-1α via β-catenin and subsequently increases mitochondrial biogenesis^51^. Although the mechanisms by which PGAM5 regulates PGC-1α, NRF1 and TFAM are still unknown, these findings suggest that PGAM5 is a mediator that stimulates mitochondrial biogenesis.

#### Regulation of mitophagy by PGAM5

Mitophagy plays a crucial role in maintaining cellular homeostasis by eliminating dysfunctional mitochondria. However, prolonged mitochondrial dysfunction may induce the excessive activation of mitophagy, resulting in pathological effects on specific organs. Parkin–PINK1-mediated mitophagy and receptor-mediated mitophagy are the principal pathways implicated in this process. According to Park et al., PGAM5 modulates PINK1–Parkin-mediated mitophagy during CCCP-induced mitochondrial dysfunction by interacting with Drp1 (ref. ^12^). In cases of mitochondrial dysfunction or oxidative stress, KEAP1 oxidation inhibits PGAM5 proteolysis. Consequently, PARL preferentially binds to PGAM5, resulting in the stable accumulation of PINK1 on the OMM. As a result, the E3 ligase activity of Parkin is activated, leading to the ubiquitylation of substrates and their binding to the ubiquitin-binding scaffold protein p62. The complex ultimately interacts with LC3 to elicit mitophagy^16,52^.

PGAM5 is reported to be involved in the activation of receptor-mediated mitophagy by regulating the dephosphorylation of the receptor FUNDC1 at Ser13, whereas CK2-mediated phosphorylation of FUNDC1 reverses the dephosphorylation of PGAM5 (refs.^8,53^). Furthermore, PGAM5 is involved in mitophagy mediated by prohibitin 2 (PHB2)^54^. During this process, PHB2 stabilizes the mitochondrial inner membrane protease PARL, preventing it from cleaving PGAM5. Intact PGAM5, in turn, stabilizes PINK1 at the OMM. This recruitment of PINK1 subsequently attracts Parkin and other mitochondrial receptors, such as NDP52, to promote mitophagy^54^. However, recent studies on endotoxemia-associated myocardial dysfunction have shown that PGAM5 inhibits mitophagy by dephosphorylating PHB2, which leads to the cytoplasmic translocation of mitochondrial PHB2 (ref. ^55^).

Collectively, these studies highlight the involvement of PGAM5 in both the Parkin–PINK1- and receptor-mediated mitophagy pathways, such as FUNDC1 and PHB2, which have competitive binding sites for PINK1, and its role as a dephosphorylating enzyme (Fig. 2b), establishing PGAM5 as a key molecule involved in mitophagy.

### PGAM5 regulates programmed cell death

#### Apoptosis mediated by PGAM5

Under specific physiological or pathological conditions, apoptosis is the process of programmed, active cell death. The anti-apoptotic BCL-2 family proteins (for example, BCL-2, BCL-x and Bcl-xL) and proapoptotic BCL-2 family proteins (for example, BAX and BAK) play crucial roles in regulating intrinsic apoptosis. Notably, PGAM5 regulates apoptosis, and a strong correlation has been observed between this process and the level of cellular stress^22,56^.

PGAM5 prevents apoptosis in nonstressed, healthy cells by inhibiting FUNDC1-mediated mitophagy and binding unphosphorylated Bcl-xL, which in turn inhibits the translocation of proapoptotic proteins to mitochondria and activates apoptotic pathways^22,57^.

**Fig. 3:**
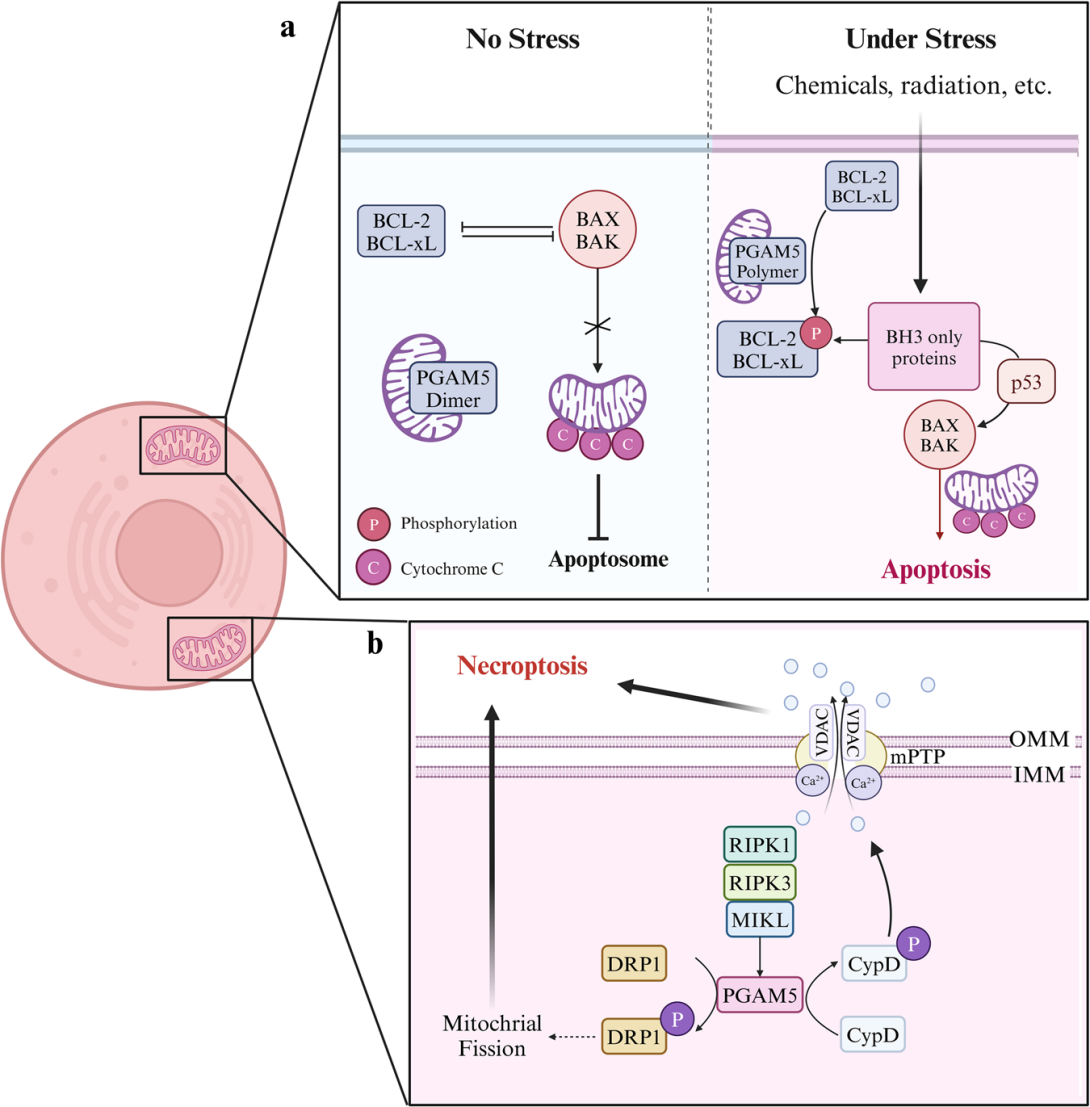
PGAM5 can mediate apoptosis and necroptosis. **a** The regulation of apoptosis by PGAM5. In unstressed cells, Bcl-xL is unphosphorylated, binds PGAM5 and exerts its anti-apoptotic function. However, Bcl-xL is phosphorylated by PGAM5 to prevent the inhibition of BAX and BAK when cells are exposed to stress, such as chemicals or radiation, ultimately leading to apoptosis. **b** PGAM5 is a downstream effector of the RIPK1/RIPK3 necroptosome that activates necroptosis through the phosphorylation of CypD and Drp1. Created with BioRender.com.

Dimeric PGAM5, which has low phosphatase activity, results in unphosphorylated Bcl-xL, which prevents apoptosis in nonstressed, healthy cells (Fig. 3a). However, stress induces the oligomerization of PGAM5 from dimers to multimers, which shifts Bcl-xL toward phosphorylation and separates Bcl-xL from proapoptotic proteins, ultimately leading to apoptosis^22,57^. In addition, PGAM5 dephosphorylates BAX in renal tubular epithelial cells and facilitates the translocation of BAX to the mitochondrial membrane^9^. The translocation of BAX to the mitochondria increases membrane permeability, decreases the Δ*Ψ*_m_, induces mitochondrial membrane permeability and promotes the release of mitochondrial cytochrome c and mtDNA into the cytoplasm, activating cellular apoptosis^9^.

However, during 5-fluorouracil therapy for HCC, PGAM5 inhibits BAX- and cytochrome C-mediated apoptotic signaling by interacting with and stabilizing Bcl-xL. This complex confers 5-fluorouracil resistance to HCC cells and functions as an anti-apoptotic factor.

In conclusion, PGAM5 participates in the apoptotic process by regulating BCL-2 family proteins via its ubiquitination and phosphorylation functions. Importantly, however, Bcl-xL possesses additional phosphorylation sites that can interact with other phosphatases^22^. Consequently, when PGAM5 binds Bcl-xL, PGAM5 is unable to modulating mitophagy to a greater extent than apoptosis.

#### Necroptosis mediated by PGAM5

Necroptosis is the necrosome-mediated mechanism of programmed cell death. PGAM5 is a direct target of RIPK3 as a downstream effector of the RIPK1/RIPK3 necroptosome^58,59^. According to He et al., PGAM5 is notably expressed in the necrotic region of hepatocytes in patients with autoimmune hepatitis^15^. PGAM5 influences ConA-induced hepatocyte necrosis and liver damage in rodents by phosphorylating Drp1 at Ser616, resulting in mitochondrial fission in hepatocytes. Similarly, Ganzleben et al. reported that during the progression of idiopathic pulmonary fibrosis, RIPK3-activated PGAM5 facilitated the activation and recruitment of Drp1 by dephosphorylating Ser637 (ref. ^38^). This process results in mitochondrial fission and dysfunction and contributes to the progression of necroptosis. Both He et al. and Ganzleben et al. proposed that the induction of Drp1-mediated mitochondrial fission was required for necroptosis. Necrotyrosine-1 inhibits necroptosis by downregulating the expression of the RIPK1–RIPK3–MLKL necroptosis complex and PGAM5, DRP1 and HMGB1 (ref.^60^). These findings further substantiate the essential function of PGAM5–Drp1 in necroptosis. Furthermore, in an ischemia–reperfusion mouse model, the upregulation of PGAM5 by RIPK3 led to an increase in the phosphorylation of CypD, which induced necroptosis in endothelial cells by increasing the opening of the mitochondrial permeability transition pore (mPTP) (Fig. 3b)^61^. These results provide additional evidence for the role of the RIPK3–PGAM5–CypD signaling pathway in necroptosis induction.

Numerous studies have shown the involvement of PGAM5 in necroptosis and its role as a target in this process. However, Moriwaki et al. reported that blocking PGAM5 in bone marrow-derived dendritic cells has no effect on necroptosis^62^. Therefore, the significance of PGAM5 in necroptosis requires further investigation and confirmation.

#### Pyroptosis mediated by PGAM5

Pyroptosis and necroptosis can result in cellular necrosis, which is characterized by the swelling of organelles, rupture of cell membranes and eventual cell lysis. However, pyroptosis, which is the primary response to infectious organisms, differs from necroptosis in terms of the mechanism of induction, with the nucleus typically remaining intact throughout the process. Pyroptosis is primarily induced by the inflammasome-mediated activation of various caspases, such as Caspase-1, and primarily manifests as the secretion of proinflammatory factors, such as mature IL-1β and IL-18, with the Nlrp3 inflammasome serving as a key regulatory signal for pyroptosis^63,64^. Kang et al. found that PGAM5 contributes to Rip3-mediated necrosis of dendritic cells and Nlrp3 inflammasome activation, suggesting that PGAM5 is involved in Rip3/Caspase-8 pathway-mediated inflammasome activation^65^. Moriwaki et al. reported that PGAM5-deficient bone marrow-derived macrophages (BMDMs) exhibited impaired IL-1β maturation, secretion and inflammasome activation. However, this impairment was not observed in BMDMs in which RIPK3 was knocked down, indicating that PGAM5 promotes inflammasome activation independent of RIPK3 (ref.^62^). The deletion of PGAM5 in microglia inhibits the accumulation of apoptosis-associated speck-like protein containing a CARD (ASC) and the subsequent activation of Caspase-1, resulting in the elimination of IL-1β secretion induced by LPS + ATP^66^. These findings highlight the role of PGAM5 in facilitating the ASC-dependent processing of IL-1β by activating the inflammasome via Caspase-1. Importantly, the involvement of PGAM5 in the Caspase-1 pathway-mediated activation of the inflammasome in microglia stimulated with LPS and ATP is independent of the RIPK3/Caspase-8 pathway.

The aforementioned studies suggest the involvement of PGAM5 in various cells via diverse mechanisms that regulate the inflammasome, and it plays an integral role in this process. Therefore, additional research is needed to elucidate the underlying mechanisms that govern the correlation between PGAM5 and the inflammasome. In addition, investigating the effects of diverse cellular phenotypes and the induction of different pathways on the functional variations observed in this process is crucial.

#### Oxeiptosis mediated by PGAM5

ROS play important roles in physiological and pathological processes. However, their sensing mechanisms and downstream signal transduction remain incompletely understood. These ROS sensors and pathways are responsible for oxidative stress-induced cell death. Oxeiptosis, a recently discovered form of cell death induced by ROS, shares characteristics with apoptosis. This newly identified cell death pathway may be prevalent in many cell types, except macrophages, which are insensitive to or even resistant to ROS-dependent cell death^62^.

**Fig. 4:**
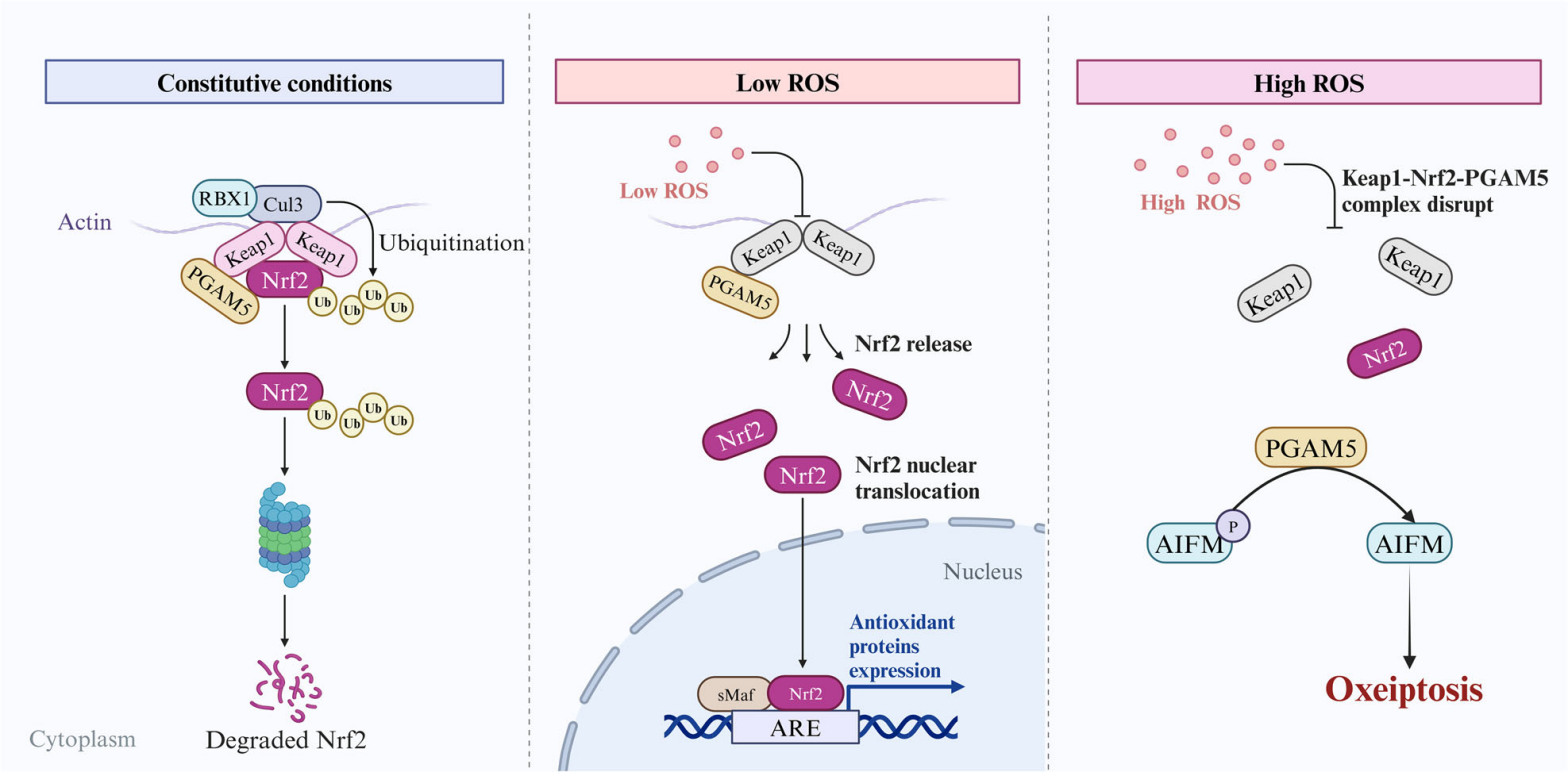
PGAM5 activates oxeiptosis in the presence of high levels of ROS. Under physiological conditions, ubiquitinated NRF2 is degraded by the proteasome; in the presence of low levels of ROS, NRF2 is released to activate the expression of antioxidant proteins, allowing the body to produce antioxidant effects. However, in the presence of high levels of ROS, the KEAP1–NRF2–PGAM5 complex dissociates, and PGAM5 binds directly to AIFM, leading to oxeiptosis. Created with BioRender.com.

Oxeiptosis depends on PGAM5 and operates independently of the characterized cell death pathways mentioned above, serving as a regulatory mechanism to limit detrimental ROS-associated inflammation^67^. The involvement of PGAM5 in oxeiptosis is closely related to its interactions with ROS sensors such as KEAP1, the antioxidant NRF2, and the proapoptotic protein AIFM1 (refs. ^68,69^). ROS inducers, such as BZL101, have been shown to induce cell death in an AIFM1-dependent manner, thereby providing a potential cancer treatment strategy^70^. These findings support the association between ROS and AIFM1. KEAP1 promotes the ubiquitination and degradation of NRF2 under normal physiological conditions. However, low ROS levels result in the release of the KEAP1–PGAM5–NRF2 complex in the cytoplasm, allowing NRF2 to accumulate and translocate into the nucleus. Within the nucleus, NRF2 activates the transcription of antioxidant genes, including *Ho1*, *Nqo1* and *Gclc*^68^, indicating the cytoprotective function of low levels of ROS, which do not induce cell death. Only high levels of ROS disrupt the KEAP1–PGAM5–NRF2 complex, leading to the dephosphorylation of AIFM1 by PGAM5 and the activation of the AIFM1-mediated cell death pathway (Fig. 4)^68^. Hence, oxeiptosis is distinguished by the dephosphorylation of AIFM1 at Ser116, which serves as a crucial factor regulating the activity of this pathway.

Pathogen infections and inflammatory factors are common causes of elevated ROS levels. As a result, different types of programmed cell death, such as oxeiptosis and necroptosis, serve as protective mechanisms against oxidative stress and actively contribute to anti-inflammatory and antiviral processes. Additionally, new evidence highlights the importance of PGAM5 in viral infections. Influenza A virus infection in mice activates RIPK3-dependent necroptosis and Caspase-8-mediated apoptosis and reduces the severity of pneumonia symptoms^71^, whereas PGAM5 deficiency in mice exacerbates the inflammatory response and viral infiltration^67^. This proinflammatory phenotype may be mediated by the PGAM5-dependent inhibition of oxeiptosis, necroptosis and apoptosis, as well as the non-PGAM5-dependent activation of programmed cell death. Different modalities of cell death influence one another and generate a compensatory response, highlighting the crucial roles of PGAM5 in regulating inflammation and infection progression and in all types of cell death.

## PGAM5 has dual abilities to exacerbate and reduce inflammation

The immune response protects against harmful agents such as viruses and bacteria. Excessive immunological reactions cause pathological damage, eventually leading to chronic inflammation and degenerative disorders such as osteoarthritis (OA). Additionally, chronic inflammation, which releases various inflammatory mediators, causes cellular and organ senescence during natural aging^72^. Senescent cells trigger immunological signals, attracting immune cells to eliminate them and further exacerbating the development of age-related inflammation. Aging and inflammation are interrelated, with reciprocal effects^73^. As a result, aging is commonly considered a type of chronic inflammation in many studies.

Numerous studies have linked mitochondrial dysfunction to the development of inflammation and aging. As an essential mitochondrial protein, PGAM5 controls inflammation and aging^13,14^. However, the biological roles of PGAM5 in various types of inflammation and aging processes are cell and organ specific (Table 3). This specificity is probably due to the differential expression of PGAM5 in distinct tissues.

**Table 3:** Cell- and organ-specific roles of PGAM5 in inflammation.

Type of inflammation/cell line	Expression of PGAM5 (compared with normal tissues/cells)	Biological processes regulated by PGAM5	Molecules involved	Signaling pathways involved	References
**Hepatic inflammation**	High	Mitochondrial fission, necroptosis	Drp1	cGAS–STING–IRF signaling pathway	^15^
**Hepatic ischemia–reperfusion injury**	Unknown	Apoptosis	RNF5, ASK1, miR-330-3p	JNK–p38 signaling pathway	^31,49,98^
**TBI**	High	Mitophagy, mitochondrial biogenesis, pyroptosis	TFAM, Caspase-1	NLRP3–Caspase-1–IL-1β signaling pathway	^66,97^
**Experimental autoimmune encephalomyelitis**	High	Necroptosis	Drp1	–	^66^
**AKI**	High	Apoptosis	BAX	cGAS–STING signaling pathway	^9^
**Diabetic tubular injury**	High	Mitochondrial fission	Sp1, Drp1	AMPK–Sp1–PGAM5 pathway	^29^
**Respiratory inflammation caused by viruses**	low	Mitochondrial dynamics, oxeiptosis	Mfn2, KEAP1, AIFM1,	RIG-I like receptor signaling pathway	^67,106^
**Acute lung injury**	Unknown	Necroptosis	RIPK3	–	^83^
**Senescent human retinal epithelial cells**	Unknown	Mitochondrial fission	Drp1	IRF–IFN-β signaling pathway, AMPK–mTOR signaling pathway	^13^
**Senescent oocytes**	High	Mitochondrial fission	Drp1	–	^14^

### Proinflammatory roles of PGAM5 in inflammation

Inflammation is a complex multistep process involving the activation of immune cells, the production of large quantities of ROS, an increase in mitochondrial membrane permeabilization and the activation of proinflammatory signaling pathways^74,75^. The inflammatory response is an essential mechanism for removing harmful factors from an organism, and this response leads to tissue repair and the restoration of normalcy, resulting in a healing outcome^76^. When an overreaction allows acute inflammation to evolve into chronic inflammation, the organism’s tissues become pathologically damaged, which in turn induces disease^76^. This process is influenced primarily by the extensive release of cellular contents into the extracellular environment, which induces extensive cell death^77^. An increasing body of evidence indicates that PGAM5 plays a pivotal role in the induction and promotion of the inflammatory response, which is mainly attributed to PGAM5-mediated mitochondrial dysfunction and programmed cell death^6^.

The overexpression of PGAM5 has been observed in some inflammatory diseases, including acute liver injury and diabetic renal tubular injury^15,29^. Increased PGAM5 results in the overactivation of Drp1-mediated mitochondrial fission, the accumulation of damaged mitochondria, a reduction in ATP production and an imbalance in mitochondrial dynamics^13,19^. Elevated levels of PGAM5 and its cofactor, Drp1, were also detected in aged granulosa cells^14^. Suppressing Drp1^Ser637^ dephosphorylation by PGAM5 deletion has been shown to inhibit the production of aberrant mitochondria, attenuate inflammatory pathological changes and notably upregulate the expression of inflammatory factors, such as IL-6 and MCP1 (ref. ^53^). Excessive mitochondrial fission is accompanied by an elevation of intracellular ROS and mitochondrial ROS levels due to the incomplete oxidation of oxygen molecules produced by oxidative phosphorylation in mitochondria following the disruption of the mitochondrial fission–fusion equilibrium^78^. The overproduction of ROS may lead to cellular and tissue damage and contribute to chronic inflammation in many neurodegenerative, cardiovascular and metabolic diseases^79–81^. As described above, the increases in ROS levels and mitochondrial dysfunction frequently affect the Δ*Ψ*_m_, mitochondrial permeability and activation of inflammasomes.

Emerging evidence indicates that necroptosis plays a crucial role in inducing proinflammatory responses. The activation of PGAM5 by the RIPK1–RIPK3–MLKL necroptotic complex is thought to promote the recruitment of Drp1 and lead to mitochondrial dysfunction^10^. Consequently, PGAM5 functions as a novel inducer of necroptosis. Receptors that mediate necroptosis through PGAM5 include Toll-like receptors and cytoplasmic nucleic acid sensors, such as RIG-I and STING^82^. This process induces the production of DAMPs, such as IFN-I and TNF, which promote necroptosis via feedback loops and the activation of innate immune cells, resulting in the production of more proinflammatory cytokines^82^. In hepatic necrosis, pretreatment of hepatic tissues with necrostatin-1 in the necro-inflamed liver notably reduces the expression of RIP1, RIP3 and MLKL, as well as PGAM5 and Drp1, leading to a marked attenuation of inflammation and injury^15^. Conversely, direct knockdown of PGAM5 results in increases in the mtDNA copy number and transcript levels at the cellular level, the normalization of mitochondrial respiration, inhibition of mitochondrial ROS production, and prevention of aberrant activation of the mPTP^53^. These changes lead to partial inhibition of mitochondrial fission and a reduction in apoptosis in cell models of injury induced by exogenous molecules, including TNF, LPS and ConA^10,15,83^. At the tissue level, silencing of PGAM5 also inhibits disease-mediated necrotic apoptosis in cardiac ischemia–reperfusion injury, acute lung injury and acute hepatitis, accompanied by improved tissue function and a reduced inflammatory response^15,84,85^. Therefore, PGAM5-mediated necroptosis is considered a potential pathogenic mechanism for many inflammatory diseases. Although several previous studies have revealed that PGAM5 is involved in necroptosis and is a direct target of RIPK3, other studies have suggested that PGAM5 is not involved in necroptosis. Knockdown of PGAM5 does not importantly affect necroptosis induced by a variety of cell death inducers, but is notably involved in pro-IL-1β processing in BMDMs^62^. These results challenge the role of PGAM5 in necroptosis.

Similarly, pyroptosis is a form of inflammatory cell necrosis that culminates in the loss of plasma membrane integrity and is induced by the activation of inflammasome sensors, including the NLR family^86^. The pyroptosis driver also detects various Pathogen-associated molecular patterns (PAMPs) and DAMPs released by microbe-infected cells or through other dysregulated cellular pathways^87^. Mitochondrial dysfunction is one of the drivers of this process, and few current studies have investigated whether it includes the dysregulation of mitochondrial dynamics and mitophagy mediated by PGAM5.

However, studies of the role of PGAM5 in pyroptosis have focused on its assembly function in inflammasomes. Downregulation of PGAM5 inhibits the assembly of Nlrp3 inflammasomes, including the Nlrp3 interaction with Caspase-1 and ASC, demonstrating that Nlrp3 inflammasome formation is indispensable for PGAM5 (ref. ^66^). Inflammasomes, which are driven by Nlrp3, act as a platform for the activation of the protein hydrolyzing enzyme Caspase-1, and active Caspase-1 can process IL-1β and IL-18 to induce their maturation^88^. In the context of chronic disease, IL-1β levels are increased in the lungs of neutrophilic asthmatic mice and neutrophilic asthmatic patients, which may lead to impaired epithelial barrier function and increased mucus production, exacerbating inflammation^68^. Recent studies have shown that the use of Pgam5 short hairpin RNA to treat traumatic brain injury (TBI) results in the abrogation of ASC-mediated Caspase-1 activation, subsequently reducing proinflammatory factor-induced IL-1β secretion, which substantiates a pivotal role for Pgam5 in the inflammatory process^66^. In addition, changes in PGAM5 are accompanied by changes in the expression of related proteins, such as Nlrp3, Caspase-1, gasdermin D (GSDMD) and IL-1β, and we speculate that PGAM5 may influence the posttranslational modifications and protein expression of these genes to regulate inflammatory responses^66^. Activating Caspase-1 via PGAM5-mediated inflammasomes also results in GSDMD cleavage, which oligomerizes and is incorporated into the plasma membrane to form pores, leading to membrane instability and cell lysis^89^. The ASCs are subsequently released from pyroptotic cells and remain stable in the tissue or circulation for several days, prolonging their proinflammatory potential^90^.

PGAM5 is a key mediator of the proinflammatory response, accelerating the progression of inflammation.

### Anti-inflammatory roles of PGAM5

However, PGAM5 also plays a dominant role in anti-inflammatory processes, demonstrating its two-sided nature.

Mitochondrial fusion and increased mitophagy lead to the maintenance of a stable mtDNA content and the degradation of damaged mitochondria, inhibiting subsequent inflammatory pathways. Following the action of CCCP, an apoptosis inducer, PGAM5 removes damaged mitochondria and protects cellular activity by activating PINK1-dependent mitophagy^26^. In the absence of PGAM5, however, PINK1 cannot receive this protection and is cleaved and degraded, inhibiting mitophagy^56^. PGAM5-mediated mitophagy also inhibits necroptosis and protects cells from proinflammatory effects^12^. Thus, PGAM5 is believed to mediate moderate mitophagy, which exerts anti-inflammatory protective effects distinct from its role in proinflammatory responses, such as neuroprotection and cardioprotection^10,12^. This difference in function may be related to the inhibition of pro-IL-1β expression and inflammasome activation by mitophagy. In addition to mitochondrial fusion and mitophagy, mitochondrial fission has also been described as having inhibitory effects on inflammation. However, as research has progressed, PGAM5-mediated mitochondrial fission has been found to be highly heterogeneous across cells. For example, cellular senescence due to reduced mitochondrial renewal caused by a lack of PGAM5 can be reversed by the overexpression of Drp1 (ref. ^13^), confirming that PGAM5–Drp1-mediated mitochondrial fission is associated with retinal pigment epithelial senescence and can play a proinflammatory role.

In addition to the two types of proinflammatory programmed cell death mediated by PGAM5, PGAM5 also mediates oxeiptosis, a form of ROS-induced cell death. Oxeiptosis has been reported to exert inflammation-suppressing effects at this stage of research^67^. Unlike its function in other death modes, PGAM5, an integral protein, in oxeiptosis is mediated by KEAP1–PGAM5–AIFM1, in which PGAM5 dephosphorylates AIFM1 (ref.^69^). HeLa cells infected with the influenza A virus accumulate intracellular ROS, mediating reduced AIFM1 phosphorylation^67^. Virus-infected mice deficient in PGAM5 accumulate notably higher levels of inflammatory cytokines, such as IL-6, CXCL1 and CCL2, leading to deeper viral infiltration and sustained exacerbation of inflammation, such as acute necrotizing endobronchial and peribronchial inflammation^67^. PGAM5 is required to regulate the inflammatory response in vivo. This protein inhibits the inflammatory response, and the lack of PGAM5 in vivo leads to a compensatory inflammatory response.

Furthermore, PGAM5 modulation of the anti-inflammatory response also inhibits the activation of proinflammatory-associated KCa3.1 potassium channels by dephosphorylating NDPK-B in CD4^+^ T cells^91^. Current studies suggest that the inhibition of this channel inhibits potassium ion (K) efflux and proinflammatory cytokine production, thereby inhibiting the progression of inflammation, such as rheumatoid arthritis and colitis^92,93^. Therefore, PGAM5 may be crucial for regulating KCa3.1, which may play a crucial role in the pathogenesis of rheumatoid arthritis and colitis in mice.

## Targeting PGAM5 for therapy

Therapies targeting PGAM5 span a diverse spectrum, reflecting its Janus-like role in inflammation and other diseases. For example, supplementation of PGAM5 with PEP-1 in hippocampal ischemic injury has been shown to reduce oxidative stress and enhance antioxidant activities (for example, glutathione peroxidase and superoxide dismutase), thereby conferring neuroprotective effects^94^.

Inhibitors targeting PGAM5 have shown promising effects in other preclinical studies. LFHP-1c, a novel PGAM5 inhibitor that protects blood–brain barrier integrity from ischemic damage, has shown neuroprotective activity in ischemic stroke, representing a potential therapeutic approach^95^. Our group has contributed similarly to the targeting of PGAM5 for the treatment of OA. A new therapeutic strategy for alleviating OA symptoms by specifically inhibiting PGAM5 expression in synovial macrophages has been proposed^96^. These findings provide a theoretical basis for early intervention in OA.

In summary, strategies targeting PGAM5 in disease treatment should consider different cell types. Furthermore, molecules that target the upstream and downstream regulation of PGAM5 expression require further investigation in future basic research and clinical trials. Similarly, techniques such as inhibitors, small interfering RNAs and microRNAs can block or overactive PGAM5 function to ameliorate disease symptoms. However, the current scientific research on PGAM5 is still in the basic research stage, and more advanced studies of PGAM5 as a potential therapeutic target are needed.

## Conclusions and prospects

PGAM5 maintains normal mitochondrial function and thus protects cellular homeostasis. Some studies have demonstrated that PGAM5 alleviates the inflammatory process and is not conducive to the senescence process^13,67^, suggesting that it functions as a positive regulator of inflammation when expressed within a specific range. However, PGAM5 expression varies in most inflammatory diseases, and its abnormal expression leads to impaired mitochondrial quality control, including mitochondrial fission^29^ and mitophagy^66,97^. Proinflammatory cytokines, chemokines and other classic inflammatory factors are upregulated in response to PGAM5 expression^31,49,98^. The exacerbated inflammation may even contribute to senescence in some cells^14^, making PGAM5 a hazardous regulator of inflammation progression.

In addition to its role in inflammation, PGAM5 has been intensively investigated in various diseases, including cancer and neurodegenerative disorders. In epileptic mouse neurons, elevated ROS levels and mitophagy are associated with PGAM5 overexpression^99^. PGAM5 exerts neuroprotective effects on CCCP-induced human neuroblastoma cells by activating mitophagy to remove damaged mitochondria^12^. This regulation is disease specific and depends on the molecular function of PGAM5 in different contexts.

In summary, an extensive body of research conducted in the past decade has provided insights into initiating and treating inflammation through potential regulatory mechanisms that govern PGAM5 levels and mitochondrial homeostasis. Given that PGAM5 is closely associated with a wide range of mitochondria-associated disorders, it could serve as a viable therapeutic target in various diseases. However, the biological function of PGAM5 is complicated and relatively challenging for clinical use. More in-depth mechanistic studies are needed in the following directions: (1) the discovery of novel metabolic mechanisms of PGAM5 opens the door to further exploration of the upstream and downstream mechanisms of oxeiptosis; (2) PGAM5 is essential for maintaining the balance between mitochondrial homeostasis and many other biological processes, such as programmed cell death, antioxidant responses and senescence. Determining whether the regulation of mitochondrial function by PGAM5 under physiological and pathological conditions is tissue/organ specific is necessary; (3) the expression of PGAM5 has diverse impacts on different disorders. Further studies are needed to characterize the related mechanisms, which can also increase the therapeutic potential of PGAM5 for a variety of diseases in different organs, particularly those that are relatively rich in mitochondria, such as the heart and liver. With more efforts being made, a more solid foundation will be built for the study of the dual properties of PGAM5. As a result, we expect to witness the development of precision and intelligent therapeutic strategies that target this protein, which will effectively treat a variety of associated types of inflammation in different organs. These strategies could be applied to other diseases, opening new possibilities for personalized medicine and precision therapy.

## Data Availability

No data were used for the research described in this Review.

## References

[1] Fulop, T. et al. Immunology of aging: the birth of inflammaging. *Clin. Rev. Allergy Immunol.* **64**, 109–122 (2023). 34536213 10.1007/s12016-021-08899-6 PMC8449217

[2] PálmaiPallag, T. & Bachrati, C. Z. Inflammation-induced DNA damage and damage-induced inflammation: a vicious cycle. *Microbes Infect.* **16**, 822–832 (2014). 25449753 10.1016/j.micinf.2014.10.001

[3] Martijn, J., Vosseberg, J., Guy, L., Offre, P. & Ettema, T. J. G. Deep mitochondrial origin outside the sampled alphaproteobacteria. *Nature* **557**, 101–105 (2018). 29695865 10.1038/s41586-018-0059-5

[4] Sliter, D. A. et al. Parkin and PINK1 mitigate STING-induced inflammation. *Nature* **561**, 258–262 (2018). 30135585 10.1038/s41586-018-0448-9 PMC7362342

[5] Forrester, S. J. et al. Mitochondrial fission mediates endothelial inflammation. *Hypertension* **76**, 267–276 (2020). 32389075 10.1161/HYPERTENSIONAHA.120.14686 PMC7289685

[6] Cheng, M. et al. PGAM5: a crucial role in mitochondrial dynamics and programmed cell death. *Eur. J. Cell Biol.* **100**, 151144 (2021). 33370650 10.1016/j.ejcb.2020.151144

[7] Dan, S. et al. PGAM5 regulates DRP1-mediated mitochondrial fission/mitophagy flux in lipid overload-induced renal tubular epithelial cell necroptosis. *Toxicol. Lett.* **372**, 14–24 (2023). 36273635 10.1016/j.toxlet.2022.10.003

[8] Chen, G. et al. A regulatory signaling loop comprising the PGAM5 phosphatase and CK2 controls receptor-mediated mitophagy. *Mol. Cell* **54**, 362–377 (2014). 24746696 10.1016/j.molcel.2014.02.034

[9] Li, J. et al. Phosphoglycerate mutase 5 initiates inflammation in acute kidney injury by triggering mitochondrial DNA release by dephosphorylating the pro-apoptotic protein Bax. *Kidney Int.* **103**, 115–133 (2023). 36089186 10.1016/j.kint.2022.08.022

[10] Nakano, H. et al. Mitochondrial protein PGAM5 regulates mitophagic protection against cell necroptosis. *PLoS ONE* **11**, e0147792 (2016). 26807733 10.1371/journal.pone.0147792 PMC4725845

[11] Zuo, W., Yan, F., Liu, Z. & Zhang, B. miR-330 regulates Drp-1 mediated mitophagy by targeting PGAM5 in a rat model of permanent focal cerebral ischemia. *Eur. Jo. Pharmacol.* **880**, 173143 (2020). 10.1016/j.ejphar.2020.173143 32360974

[12] Park, Y. S., Choi, S. E. & Koh, H. C. PGAM5 regulates PINK1/Parkin-mediated mitophagy via DRP1 in CCCP-induced mitochondrial dysfunction. *Toxicol. Lett.* **284**, 120–128 (2018). 29241732 10.1016/j.toxlet.2017.12.004

[13] Yu, B. et al. Mitochondrial phosphatase PGAM5 modulates cellular senescence by regulating mitochondrial dynamics. *Nat. Commun.* **11**, 2549 (2020). 32439975 10.1038/s41467-020-16312-7 PMC7242393

[14] Li, C., Lin, L., Tsai, H., Wen, Z. & Tsui, K. Phosphoglycerate mutase family member 5 maintains oocyte quality via mitochondrial dynamic rearrangement during aging. *Aging Cell* **21**, e13546 (2022). 34995407 10.1111/acel.13546 PMC8844125

[15] He, G. W. et al. PGAM5-mediated programmed necrosis of hepatocytes drives acute liver injury. *Gut* **66**, 716–723 (2017). 27566130 10.1136/gutjnl-2015-311247

[16] Sekine, S. et al. Rhomboid protease PARL mediates the mitochondrial membrane potential loss-induced cleavage of PGAM5. *J. Biol. Chem.* **287**, 34635–34645 (2012). 22915595 10.1074/jbc.M112.357509 PMC3464569

[17] Lo, S. & Hannink, M. PGAM5 tethers a ternary complex containing Keap1 and Nrf2 to mitochondria. *Exp. Cell Res.* **314**, 1789–1803 (2008). 18387606 10.1016/j.yexcr.2008.02.014 PMC2409987

[18] Takeda, K. et al. Mitochondrial phosphoglycerate mutase 5 uses alternate catalytic activity as a protein serine/threonine phosphatase to activate ASK1. *Proc. Natl Acad. Sci. USA* **106**, 12301–12305 (2009). 19590015 10.1073/pnas.0901823106 PMC2718331

[19] Chen, Y. et al. Phosphoglycerate mutase 5 knockdown alleviates neuronal injury after traumatic brain injury through Drp1-mediated mitochondrial dysfunction. *Antioxid. Redox Signal.* **34**, 154–170 (2021). 32253918 10.1089/ars.2019.7982

[20] Wang, Z., Jiang, H., Chen, S., Du, F. & Wang, X. The mitochondrial phosphatase PGAM5 functions at the convergence point of multiple necrotic death pathways. *Cell* **148**, 228–243 (2012). 22265414 10.1016/j.cell.2011.11.030

[21] Kang, Y. J. et al. Regulation of NKT cell-mediated immune responses to tumours and liver inflammation by mitochondrial PGAM5–Drp1 signalling. *Nat. Commun.* **6**, 8371 (2015). 26381214 10.1038/ncomms9371 PMC4576739

[22] Ma, K. et al. Dynamic PGAM5 multimers dephosphorylate BCL-xL or FUNDC1 to regulate mitochondrial and cellular fate. *Cell Death Differ.* **27**, 1036–1051 (2019). 31367011 10.1038/s41418-019-0396-4 PMC7206082

[23] Sugo M. et al. Syntaxin 17 regulates the localization and function of PGAM5 in mitochondrial division and mitophagy. *EMBO J.*10.15252/embj.201798899 (2018). 10.15252/embj.201798899 PMC6213275 30237312

[24] Saita, S., Shirane, M. & Nakayama, K. I. Selective escape of proteins from the mitochondria during mitophagy. *Nat. Commun.* **4**, 1410 (2013). 23361001 10.1038/ncomms2400

[25] Yamaguchi, A. et al. Cleaved PGAM5 is released from mitochondria depending on proteasome-mediated rupture of the outer mitochondrial membrane during mitophagy. *J. Biochem.* **165**, 19–25 (2019). 30247576 10.1093/jb/mvy077

[26] Baba, T. et al. Cleaved PGAM5 dephosphorylates nuclear serine/arginine-rich proteins during mitophagy. *Biochim. Biophys. Acta Mol. Cell Res.* **1868**, 119045 (2021). 33872670 10.1016/j.bbamcr.2021.119045

[27] Chaikuad, A. et al. Structures of PGAM5 provide insight into active site plasticity and multimeric assembly. *Structure* **25**, 1089–1099.e3 (2017). 28648608 10.1016/j.str.2017.05.020 PMC5501728

[28] Ruiz, K. et al. Functional role of PGAM5 multimeric assemblies and their polymerization into filaments. *Nat. Commun.* **10**, 531 (2019). 30705304 10.1038/s41467-019-08393-w PMC6355839

[29] Liu, X. et al. Empagliflozin improves diabetic renal tubular injury by alleviating mitochondrial fission via AMPK/Sp1/PGAM5 pathway. *Metabolism* **111**, 154334 (2020). 32777444 10.1016/j.metabol.2020.154334

[30] Zhu, Y. et al. Dynamic regulation of ME1 phosphorylation and acetylation affects lipid metabolism and colorectal tumorigenesis. *Mol. Cell* **77**, 138–149.e5 (2020). 31735643 10.1016/j.molcel.2019.10.015

[31] Ding, M. et al. E3 ubiquitin ligase ring finger protein 5 protects against hepatic ischemia reperfusion injury by mediating phosphoglycerate mutase family member 5 ubiquitination. *Hepatology* **76**, 94–111 (2022). 34735734 10.1002/hep.32226 PMC9303746

[32] Liu, S. et al. The E3 ubiquitin ligase MARCH2 protects against myocardial ischemia–reperfusion injury through inhibiting pyroptosis via negative regulation of PGAM5/MAVS/NLRP3 axis. Cell Discov. 10.1038/s41421-023-00622-3 (2024). 10.1038/s41421-023-00622-3 PMC10897310 38409220

[33] Lenhausen, A. M. et al. Apoptosis inducing factor binding protein PGAM5 triggers mitophagic cell death that is inhibited by the ubiquitin ligase activity of X-linked inhibitor of apoptosis. *Biochemistry* **55**, 3285–3302 (2016). 27218139 10.1021/acs.biochem.6b00306

[34] Lysyk, L., Brassard, R., Touret, N. & Lemieux, M. J. PARL protease: a glimpse at intramembrane proteolysis in the inner mitochondrial membrane. *J. Mol. Biol.* **432**, 5052–5062 (2020). 32320686 10.1016/j.jmb.2020.04.006

[35] Wai, T. et al. The membrane scaffold SLP2 anchors a proteolytic hub in mitochondria containing PARL and the i-AAA protease YME1L. *EMBO Rep.* **17**, 1844–1856 (2016). 27737933 10.15252/embr.201642698 PMC5283581

[36] Chen, W. et al. TIPE3 represses head and neck squamous cell carcinoma progression via triggering PGAM5 mediated mitochondria dysfunction. *Cell Death Dis.* **14**, 251 (2023). 37024453 10.1038/s41419-023-05775-3 PMC10079926

[37] Garone, C. et al. Mitochondrial dynamics: overview of molecular mechanisms. *Essays Biochem.* **62**, 341–360 (2018). 30030364 10.1042/EBC20170104 PMC6056715

[38] Ganzleben, I. et al. PGAM5 is a key driver of mitochondrial dysfunction in experimental lung fibrosis. *Cell. Mol. Life Sci.* **76**, 4783–4794 (2019). 31168659 10.1007/s00018-019-03133-1 PMC11105634

[39] Tresse, E. et al. IFN‐β rescues neurodegeneration by regulating mitochondrial fission via STAT5, PGAM5, and Drp1. *EMBO J.* **40**, e106868 (2021). 33913175 10.15252/embj.2020106868 PMC8167366

[40] Zorov, D. et al. Lessons from the discovery of mitochondrial fragmentation (fission): a review and update. *Cells* **8**, 175 (2019). 30791381 10.3390/cells8020175 PMC6406845

[41] Reddy, P. H. Increased mitochondrial fission and neuronal dysfunction in Huntington’s disease: implications for molecular inhibitors of excessive mitochondrial fission. *Drug Discov. Today* **19**, 951–955 (2014). 24681059 10.1016/j.drudis.2014.03.020 PMC4191657

[42] Chan, D. C. Mitochondrial dynamics and its involvement in disease. *Ann. Rev. Pathol.* **15**, 235–259 (2020). 31585519 10.1146/annurev-pathmechdis-012419-032711

[43] Giacomello, M., Pyakurel, A., Glytsou, C. & Scorrano, L. The cell biology of mitochondrial membrane dynamics. *Nat. Rev. Mol. Cell Biol.* **21**, 204–224 (2020). 32071438 10.1038/s41580-020-0210-7

[44] Chen, H. & Chan, D. C. Physiological functions of mitochondrial fusion. *Ann. N. Y. Acad. Sci.* **1201**, 21–25 (2010). 20649534 10.1111/j.1749-6632.2010.05615.x

[45] Nag, S. et al. PGAM5 is an MFN2 phosphatase that plays an essential role in the regulation of mitochondrial dynamics. *Cell Rep.* **42**, 112895 (2023). 37498743 10.1016/j.celrep.2023.112895

[46] Suliman, H. B., Piantadosi, C. A. & Mattson, M. P. Mitochondrial quality control as a therapeutic target. *Pharmacol. Rev.* **68**, 20–48 (2016). 26589414 10.1124/pr.115.011502 PMC11060432

[47] Bernkopf, D. B. et al. Pgam5 released from damaged mitochondria induces mitochondrial biogenesis via Wnt signaling. *J. Cell Biol.* **217**, 1383–1394 (2018). 29438981 10.1083/jcb.201708191 PMC5881504

[48] Denk, D. et al. Expansion of T memory stem cells with superior anti-tumor immunity by Urolithin A-induced mitophagy. *Immunity* **55**, 2059–2073.e8 (2022). 36351375 10.1016/j.immuni.2022.09.014

[49] Hong, J. & Lee, S. Heme oxygenase-1 protects liver against ischemia/reperfusion injury via phosphoglycerate mutase family member 5-mediated mitochondrial quality control. *Life Sci.* **200**, 94–104 (2018). 29524517 10.1016/j.lfs.2018.03.017

[50] Friedman, J. R. & Nunnari, J. Mitochondrial form and function. *Nature* **505**, 335–343 (2014). 24429632 10.1038/nature12985 PMC4075653

[51] Krantz, S. et al. Mitophagy mediates metabolic reprogramming of induced pluripotent stem cells undergoing endothelial differentiation. *J. Biol. Chem.* **297**, 101410 (2021). 34785214 10.1016/j.jbc.2021.101410 PMC8661016

[52] Zeb, A. et al. A novel role of KEAP1/PGAM5 complex: ROS sensor for inducing mitophagy. *Redox Biol.* **48**, 102186 (2021). 34801863 10.1016/j.redox.2021.102186 PMC8607199

[53] Zhu, H. et al. Phosphoglycerate mutase 5 exacerbates cardiac ischemia–reperfusion injury through disrupting mitochondrial quality control. *Redox Biol.* **38**, 101777 (2021). 33166869 10.1016/j.redox.2020.101777 PMC7658715

[54] Yan, C. et al. PHB2 (prohibitin 2) promotes PINK1–PRKN/Parkin-dependent mitophagy by the PARL–PGAM5–PINK1 axis. *Autophagy* **16**, 419–434 (2019). 31177901 10.1080/15548627.2019.1628520 PMC6999623

[55] Cai, C. et al. Pgam5-mediated PHB2 dephosphorylation contributes to endotoxemia-induced myocardial dysfunction by inhibiting mitophagy and the mitochondrial unfolded protein response. *Int. J. Biol. Sci.* **19**, 4657–4671 (2023). 37781037 10.7150/ijbs.85767 PMC10535708

[56] Lu, W. et al. Genetic deficiency of the mitochondrial protein PGAM5 causes a Parkinson’s-like movement disorder. *Nat. Commun.* **5**, 4930 (2014). 25222142 10.1038/ncomms5930 PMC4457367

[57] Lessene, G., Czabotar, P. E. & Colman, P. M. BCL-2 family antagonists for cancer therapy. *Nat. Rev. Drug Discov.* **7**, 989–1000 (2008). 19043450 10.1038/nrd2658

[58] Murphy, JamesM. et al. The pseudokinase MLKL mediates necroptosis via a molecular switch mechanism. *Immunity* **39**, 443–453, 10.1016/j.immuni.2013.06.018 (2013). 24012422 10.1016/j.immuni.2013.06.018

[59] Marshall, K. D. & Baines, C. P. Necroptosis: is there a role for mitochondria? *Front. Physiol.* **5**, 323 (2014). 25206339 10.3389/fphys.2014.00323 PMC4144201

[60] Xu, Q. et al. Necroptosis underlies hepatic damage in a piglet model of lipopolysaccharide-induced sepsis. *Front. Immunol.* **12**, 633830 (2021). 33777021 10.3389/fimmu.2021.633830 PMC7994362

[61] Zhou, H. et al. Inhibitory effect of melatonin on necroptosis via repressing the Ripk3–PGAM5–CypD–mPTP pathway attenuates cardiac microvascular ischemia–reperfusion injury. *J. Pineal Res.* **65**, e12503 (2018). 29770487 10.1111/jpi.12503

[62] Moriwaki, K. et al. The mitochondrial phosphatase PGAM5 is dispensable for necroptosis but promotes inflammasome activation in macrophages. *J. Immunol.* **196**, 407–415 (2016). 26582950 10.4049/jimmunol.1501662 PMC4684958

[63] Luo, B. et al. Using a gene network of pyroptosis to quantify the responses to immunotherapy and prognosis for neuroblastoma patients. *Front. Immunol.* **13**, 845757 (2022). 35401536 10.3389/fimmu.2022.845757 PMC8987018

[64] O’Brien, W. T. et al. The NLRP3 inflammasome in traumatic brain injury: potential as a biomarker and therapeutic target. *J. Neuroin flammation* **17**, 104 (2020). 10.1186/s12974-020-01778-5 PMC7137518 32252777

[65] Kang, T., Yang, S., Toth, B., Kovalenko, A. & Wallach, D. Caspase-8 blocks kinase RIPK3-mediated activation of the NLRP3 inflammasome. *Immunity* **38**, 27–40 (2013). 23260196 10.1016/j.immuni.2012.09.015

[66] Chen, Y. et al. Downregulation of phosphoglycerate mutase 5 improves microglial inflammasome activation after traumatic brain injury. *Cell Death Discov.* **7**, 290 (2021). 34642327 10.1038/s41420-021-00686-8 PMC8511105

[67] Holze, C. et al. Oxeiptosis, a ROS-induced caspase-independent apoptosis-like cell-death pathway. *Nat. Immunol.* **19**, 130–140 (2017). 29255269 10.1038/s41590-017-0013-y PMC5786482

[68] Sokolowska, M. et al. Acute respiratory barrier disruption by ozone exposure in mice. *Front. Immunol.* **10**, 2169 (2019). 31608051 10.3389/fimmu.2019.02169 PMC6758598

[69] Kang, P. et al. Oxeiptosis: a novel pathway of melanocytes death in response to oxidative stress in vitiligo. *Cell Death Discov.* **8**, 70 (2022). 35177586 10.1038/s41420-022-00863-3 PMC8854565

[70] Marconett, C. N. et al. BZL101, a phytochemical extract from the *Scutellaria barbata* plant, disrupts proliferation of human breast and prostate cancer cells through distinct mechanisms dependent on the cancer cell phenotype. *Cancer Biol. Ther.* **10**, 397–405 (2014). 10.4161/cbt.10.4.12424 PMC3040855 20574166

[71] Nogusa, S. et al. RIPK3 activates parallel pathways of MLKL-driven necroptosis and FADD-mediated apoptosis to protect against influenza A virus. *Cell Host Microbe* **20**, 13–24 (2016). 27321907 10.1016/j.chom.2016.05.011 PMC5026823

[72] Li, Y., Klein, C. & Kotlarz, D. Dysregulation of cell death in human chronic inflammation. *Cold Spring Harb. Perspect. Biol.* **12**, a037036 (2020). 31843991 10.1101/cshperspect.a037036 PMC7328459

[73] Fülöp, T., Larbi, A. & Witkowski, JacekM. Human inflammaging. *Gerontology* **65**, 495–504 (2019). 31055573 10.1159/000497375

[74] Zhao, M. et al. Mitochondrial ROS promote mitochondrial dysfunction and inflammation in ischemic acute kidney injury by disrupting TFAM-mediated mtDNA maintenance. *Theranostics* **11**, 1845–1863 (2021). 33408785 10.7150/thno.50905 PMC7778599

[75] Vringer, E. & Tait, S. W. G. Mitochondria and cell death-associated inflammation. *Cell Death Differ.* **30**, 304–312 (2023). 36447047 10.1038/s41418-022-01094-w PMC9950460

[76] Eming, S. A., Wynn, T. A. & Martin, P. Inflammation and metabolism in tissue repair and regeneration. *Science* **356**, 1026–1030 (2017). 28596335 10.1126/science.aam7928

[77] Anderton, H., Wicks, I. P. & Silke, J. Cell death in chronic inflammation: breaking the cycle to treat rheumatic disease. *Nat. Rev. Rheumatol.* **16**, 496–513 (2020). 32641743 10.1038/s41584-020-0455-8

[78] Jiang, Y. et al. Caveolin-1 controls mitochondrial damage and ROS production by regulating fission–fusion dynamics and mitophagy. *Redox Biol.* **52**, 102304 (2022). 35413643 10.1016/j.redox.2022.102304 PMC9018165

[79] Li, S. et al. PGAM5 expression levels in heart failure and protection ROS-induced oxidative stress and ferroptosis by Keap1/Nrf2. *Clin. Exp. Hypertens.* **45**, 2162537 (2023). 36780919 10.1080/10641963.2022.2162537

[80] Kalyanaraman, B., Cheng, G. & Hardy, M. Gut microbiome, short-chain fatty acids, alpha-synuclein, neuroinflammation, and ROS/RNS: relevance to Parkinson’s disease and therapeutic implications. *Redox Biol.* **71**, 103092 (2024). 38377788 10.1016/j.redox.2024.103092 PMC10891329

[81] Forrester, S. J., Kikuchi, D. S., Hernandes, M. S., Xu, Q. & Griendling, K. K. Reactive oxygen species in metabolic and inflammatory signaling. *Circ. Res.* **122**, 877–902 (2018). 29700084 10.1161/CIRCRESAHA.117.311401 PMC5926825

[82] Pasparakis, M. & Vandenabeele, P. Necroptosis and its role in inflammation. *Nature* **517**, 311–320 (2015). 25592536 10.1038/nature14191

[83] Zhu, P. et al. NR4A1 promotes LPS-induced acute lung injury through inhibition of Opa1-mediated mitochondrial fusion and activation of PGAM5-related necroptosis. *Oxid. Med. Cell. Longev.* **2022**, 1–18 (2022). 10.1155/2022/6638244 PMC8881136 35222801

[84] Yang, C. et al. Mitochondrial phosphatase PGAM5 regulates Keap1-mediated Bcl-xL degradation and controls cardiomyocyte apoptosis driven by myocardial ischemia/reperfusion injury. *In Vitro Cell. Dev. Biol. Anim.* **53**, 248–257 (2016). 27815660 10.1007/s11626-016-0105-2

[85] Liu, G. & Qian, M. miR-21-5p suppresses mitophagy to alleviate hyperoxia-induced acute lung injury by directly targeting PGAM5. *Biomed Res. Int.* **2020**, 4807254 (2020). 33681349 10.1155/2020/4807254 PMC7907750

[86] Bertheloot, D. & Latz, E. Necroptosis, pyroptosis and apoptosis: an intricate game of cell death. *Cell. Mol. Immunol.* **18**, 1106–1121 (2021). 33785842 10.1038/s41423-020-00630-3 PMC8008022

[87] Rao, Z. et al. Pyroptosis in inflammatory diseases and cancer. *Theranostics* **12**, 4310–4329 (2022). 35673561 10.7150/thno.71086 PMC9169370

[88] Fu, J. & Wu, H. Structural mechanisms of NLRP3 inflammasome assembly and activation. *Annu. Rev. Immunol.* **41**, 301–316 (2023). 36750315 10.1146/annurev-immunol-081022-021207 PMC10159982

[89] McKenzie, B. A. et al. Caspase-1 inhibition prevents glial inflammasome activation and pyroptosis in models of multiple sclerosis. *Proc. Natl Acad. Sci. USA* **115**, E6065–e6074 (2018). 29895691 10.1073/pnas.1722041115 PMC6042136

[90] Hoss, F., Rodriguez-Alcazar, J. F. & Latz, E. Assembly and regulation of ASC specks. *Cell. Mol. Life Sci.* **74**, 1211–1229 (2016). 27761594 10.1007/s00018-016-2396-6 PMC11107573

[91] Panda, S. et al. Identification of PGAM5 as a mammalian protein histidine phosphatase that plays a central role to negatively regulate CD4^+^ T cells. *Mol. Cell* **63**, 457–469 (2016). 27453048 10.1016/j.molcel.2016.06.021 PMC5677525

[92] Zeng, B. et al. Dextran sodium sulfate potentiates NLRP3 inflammasome activation by modulating the KCa3.1 potassium channel in a mouse model of colitis. *Cell. Mol. Immunol.* **19**, 925–943 (2022). 35799057 10.1038/s41423-022-00891-0 PMC9338299

[93] Friebel, K., Schönherr, R., Kinne, R. W. & Kunisch, E. Functional role of the KCa3.1 potassium channel in synovial fibroblasts from rheumatoid arthritis patients. *J. Cell. Physiol.* **230**, 1677–1688 (2015). 25545021 10.1002/jcp.24924

[94] Jung, H. Y. et al. The neuroprotective effects of phosphoglycerate mutase 5 are mediated by decreasing oxidative stress in HT22 hippocampal cells and gerbil hippocampus. *Neurochem. Int.* **157**, 105346 (2022). 35513204 10.1016/j.neuint.2022.105346

[95] Gao, C. et al. A novel PGAM5 inhibitor LFHP-1c protects blood–brain barrier integrity in ischemic stroke. *Acta Pharm. Sin. B* **11**, 1867–1884 (2021). 34386325 10.1016/j.apsb.2021.01.008 PMC8343116

[96] Liu, Y. et al. Targeted knockdown of PGAM5 in synovial macrophages efficiently alleviates osteoarthritis. *Bone Res.* **12**, 15 (2024). 38433252 10.1038/s41413-024-00318-8 PMC10909856

[97] Liang, M., Lu, T. & Chen, L. Timely expression of PGAM5 and its cleavage control mitochondrial homeostasis during neurite re-growth after traumatic brain injury. *Cell Biosci.* **13**, 96 (2023). 37221611 10.1186/s13578-023-01052-0 PMC10207772

[98] Sun, X., Zhang, Y., Xi, S., Ma, L. & Li, S. MiR‐330‐3p suppresses phosphoglycerate mutase family member 5‐inducted mitophagy to alleviate hepatic ischemia‐reperfusion injury. *J. Cell. Biochem.* **120**, 4255–4267 (2018). 30269356 10.1002/jcb.27711

[99] Zhong, F. et al. The inhibition of PGAM5 suppresses seizures in a kainate-induced epilepsy model via mitophagy reduction. *Front. Mol. Neurosci.* **15**, 1047801 (2022). 36618822 10.3389/fnmol.2022.1047801 PMC9813404

[100] Cheng, J. et al. High PGAM5 expression induces chemoresistance by enhancing Bcl-xL-mediated anti-apoptotic signaling and predicts poor prognosis in hepatocellular carcinoma patients. *Cell Death Dis.* **9**, 991 (2018). 30250224 10.1038/s41419-018-1017-8 PMC6155280

[101] Wang, Y. et al. AMP-activated protein kinase protects against necroptosis via regulation of Keap1–PGAM5 complex. *Int. J. Cardiol.* **259**, 153–162 (2018). 29579593 10.1016/j.ijcard.2018.01.036 PMC5873603

[102] Yu, Y. Q. et al. PGAM5-MAVS interaction regulates TBK1/IRF3 dependent antiviral responses. *Sci. Rep.* **10**, 8323 (2020). 32433485 10.1038/s41598-020-65155-1 PMC7239892

[103] Tang, Q. et al. Trehalose ameliorates oxidative stress-mediated mitochondrial dysfunction and ER stress via selective autophagy stimulation and autophagic flux restoration in osteoarthritis development. *Cell Death Dis.* **8**, e3081–e3081 (2017). 28981117 10.1038/cddis.2017.453 PMC5680575

[104] Lo, S. & Hannink, M. PGAM5, a Bcl-XL-interacting protein, is a novel substrate for the redox-regulated Keap1-dependent ubiquitin ligase complex. *J. Biol. Chem.* **281**, 37893–37903 (2006). 17046835 10.1074/jbc.M606539200

[105] Wu, H. et al. The BCL2L1 and PGAM5 axis defines hypoxia-induced receptor-mediated mitophagy. *Autophagy* **10**, 1712–1725 (2014). 25126723 10.4161/auto.29568 PMC4198357

[106] Yang, Z. et al. The expression of IFN-β is suppressed by the viral 3D polymerase via its impact on PGAM5 expression during enterovirus D68 infection. *Virus Res.* **304**, 198549 (2021). 34425164 10.1016/j.virusres.2021.198549

